# Letter to the editor on: Oral care and nosocomial pneumonia: a systematic review. einstein (São Paulo). 2015;13(2):290-6.

**DOI:** 10.1590/S1679-45082015CE3454

**Published:** 2015

**Authors:** Marcos Roberto Tovani-Palone

**Affiliations:** Hospital de Reabilitação de Anomalias Craniofaciais da Universidade de São Paulo, Bauru, SP, Brazil.

Dear Editor,

I appreciated the study “Oral care and nosocomial pneumonia: a systematic review”,^1^ which emphasizes the importance of oral care in critical patients, with a particular impact on reducing the cases of nosocomial pneumonia. Currently, the dental surgeons make an effort to implement this type of preventive action in intensive care units. However, I would like to take this opportunity and highlight that preventive oral care should also be delivered to other inpatients at different units. The *Hospital de Reabilitação de Anomalias Craniofaciais da Universidade de São Paulo* (HRAC/USP) already provides this multidisciplinary service, by giving orientations about oral care and follow-up to all inpatients.^2^ It is important to bear in mind that the use of antibiotics associated with inappropriate oral care may trigger postoperative complications, considering the existence of a continuous flow of microorganisms from the external environment to the oral cavity.^3,4^
