# Supplementary material for: Letter to the editor on: Oral care and nosocomial pneumonia: a systematic review. einstein (São Paulo). 2015;13(2):290-6
Source: Einstein (Sao Paulo). 2015 Jul-Sep;13(3):479. doi: 10.1590/S1679-45082015CE3454 (PMC4943802; doi:10.1590/S1679-45082015CE3454)
Supplement: Translation

# Translation: Carta ao editor para: Cuidados bucais e pneumonia nosocomial: revisão sistemática. einstein (São Paulo). 2015;13(2):290-6.

**Authors:** Marcos Roberto Tovani-Palone

**Affiliations:** Hospital de Reabilitação de Anomalias Craniofaciais da Universidade de São Paulo, Bauru, SP, Brasil.

Estimado Sr. Editor,

Apreciei o estudo “Cuidados bucais e pneumonia nosocomial: revisão sistemática”,^1^ que reforça a importância da aplicação dos cuidados de higiene bucal em pacientes críticos, com impacto sobretudo na diminuição da ocorrência de pneumonia nosocomial. Destaca-se que atualmente há uma grande ênfase por parte da classe odontológica com vistas à implementação desse tipo de serviço preventivo em unidades de terapia intensiva. No entanto, oportunamente, gostaria de salientar que serviços preventivos complementares de higiene bucal também deveriam ser estendidos para os outros internos das unidades hospitalares. Um exemplo é o que já ocorre no Hospital de Reabilitação de Anomalias Craniofaciais da Universidade de São Paulo (HRAC/USP), onde um serviço com âmbito coletivo proporciona instruções e acompanhamento para realização de higiene bucal em todos os pacientes internados;^2^ posto que o uso de antibióticos e condições inadequadas de higienização bucal diante do fluxo contínuo de microrganismos, via meio externo-cavidade bucal, podem desencadear principalmente complicações pós-cirúrgicas.^3,4^
